# Preoperative versus Postoperative Rectus Sheath Block for Acute Postoperative Pain Relief after Laparoscopic Cholecystectomy: A Randomized Controlled Study

**DOI:** 10.3390/jcm8071018

**Published:** 2019-07-11

**Authors:** Hye-Won Jeong, Chan Sik Kim, Kyu Taek Choi, Sung-Moon Jeong, Doo-Hwan Kim, Jong-Hyuk Lee

**Affiliations:** 1 Department of Anesthesiology and Pain Medicine, International St. Mary’s Hospital, Catholic Kwandong University College of Medicine, Incheon 22711, Korea; 2 Department of Anesthesiology and Pain Medicine, Asan Medical Center, University of Ulsan College of Medicine, Seoul 05505, Korea

**Keywords:** preemptive analgesia, rectus sheath block, laparoscopic cholecystectomy

## Abstract

Background: Pain after laparoscopic cholecystectomy (LC) is multifactorial and usually not effectively treated. Rectus sheath block (RSB) has been proven to reduce the pain from midline abdominal incision and laparoscopic surgery. We investigated the preemptive analgesic effect of RSB after LC. Methods: In this prospective, randomized, single-center trial, 200 patients undergoing LC were randomized into preoperative RSB (pre-RSB) or postoperative RSB (post-RSB) group. An ultrasound-guided RSB was performed before skin incision in the pre-RSB group or after skin closure in the post-RSB group. The primary outcome was total rescue analgesic consumption at 24 h post-surgery. The secondary outcomes were cumulated rescue analgesic consumption and postoperative pain measured by numerical rating scale (NRS) at 0, 1, 2, 6, 9, 18, and 24 h post-surgery. Results: Total rescue analgesic consumption at 24 h post-surgery was significantly lower in the pre-RSB group than in the post-RSB group (*p* = 0.020). The cumulated rescue analgesic consumption was significantly lower in the pre-RSB group than in the post-RSB group at 1 h (*p* = 0.023), 9 h (*p* = 0.020) and 18 h (*p* = 0.002) post-surgery. NRS was significantly lower in the pre-RSB group than in the post-RSB group at 0 h post-surgery (*p* = 0.023). Conclusion: The pre-RSB reduced the analgesic requirements in patients undergoing LC compared with the post-RSB.

## 1. Introduction

Cholecystectomy is one of the most commonly performed elective surgical procedures and laparoscopic cholecystectomy (LC) has become the method of choice in most benign conditions [1]. Compared with open cholecystectomy, LC has been considered to be a minimally invasive surgery with less postoperative pain [2]. However, in the immediate postoperative period, LC causes moderate to severe postoperative pain [3,4], which is usually not sufficiently managed, resulting in patient discomfort and delayed recovery [4,5].

Pain after LC is multifactorial, as incisional pain, visceral pain, and referred shoulder pain are all implicated. The overall pain is most intense on the day of LC with the incisional pain predominating over the visceral pain [4,6]. To provide procedure-specific pain management, various multimodal analgesic strategies for patients undergoing LC have been tried [7,8,9]; administration of NSAIDs, COX-2 inhibitors, dexamethasone, and port site-local anesthetic infiltration (LAI) are recommended [8,9]. Abdominal truncal blocks have been recommended as a part of multimodal analgesia in laparoscopic abdominal surgery [10,11]. However, the analgesic effect of transversus abdominis plane (TAP) block is debatable in LC [9,12]. Although few data are available on RSB in LC, considering the analgesic effects of RSB in laparoscopic surgery and umbilical surgery [13,14,15,16,17], RSB may be effective for pain relief after LC.

Preemptive analgesia is an antinociceptive treatment that prevents the establishment of central sensitization caused by incisional and inflammatory injuries, which amplifies postoperative pain [18,19]. Sufficient blockade of perioperative nociceptive input may reduce pathologic hypersensitivity, thereby improving the pain after surgery [18,19]. Preemptive analgesia has been studied in diverse surgical settings; however, the results of experimental studies on its effects are still controversial [18,20,21].

In the present study, we hypothesized that preoperative RSB (pre-RSB) may lessen the deleterious impact of intraoperative and early postoperative noxious input, thus better preventing the induction of central sensitization and pathologic pain than postoperative RSB (post-RSB). We aimed to investigate the preemptive effect of preoperative RSB on the pain after LC.

## 2. Materials and Methods

### 2.1. Patients

This single-center, prospective, randomized, single-blind study was conducted at Asan Medical Center in Seoul, Republic of Korea. The study protocol was approved by the Institutional Review Board of Asan Medical Center (2017-0301), and written informed consent was received from all participants in the study. This study was registered at ClinicalTrials.gov (NCT03413280). Adult patients scheduled for elective LC were considered eligible for the study. Patients were enrolled if they were aged 20–80 years with American Society of Anesthesiologists physical status class ≤2. We excluded the patients who (1) declined to participate; (2) had used an anticoagulant; (3) were suspected to have severe adhesions; (4) were allergic to local anesthetics; (5) had serious neurological or psychiatric disorders; (6) were pregnant or breastfeeding; and (7) were scheduled to undergo a single-port LC.

### 2.2. Randomization

Using a computer-generated randomization sheet, the enrolled patients were randomly assigned to either the pre-RSB or post-RSB group. For the random allocation of participants, the web-based randomization software (Random Allocation Software version 1.0, Isfahan University of Medical Sciences, Isfahan, Iran) was used with random block sizes of 4 and an allocation ratio of 1:1. The allocation sequence was concealed from the first investigator enrolling and assessing participants in opaque, sealed, and sequentially numbered envelopes, which were given to the intervention staff who conducted anesthesia and ultrasound-guided RSB and intercostal nerve block (ICNB). A second investigator, who was blinded to treatment allocation, administered analgesics at the post-anesthesia care unit (PACU) and general ward during 24 h after surgery and assessed postoperative outcomes. Although treatment allocation was un-blinded to the intervention staff, it was kept blinded to the second investigators and participants.

### 2.3. Surgical Technique

The same team of experienced laparoscopic surgeons (carried out more than 300 LCs) performed all operations. All patients underwent standard procedures for LC. Three trocars were inserted below the xiphoid process (5 mm), right costal arch (5 mm), and umbilicus (10 mm). Via the infraumbilical incision, a camera port was inserted, and the gallbladder was retracted. Pneumoperiotoneum was created and maintained using carbon dioxide insufflation with intraperitoneal pressure of 12 mmHg. LC was conducted in the supine position with a 30° reverse Trendelenburg position.

### 2.4. Anesthesia and Analgesia

In the operating room, all patients were routinely monitored using electrocardiography, non-invasive blood pressure measurement, and pulse oximetry. Anesthesia was induced using propofol (2 mg/kg), rocuronium (0.6 mg/kg), and remifentanil continuous infusion using a target-controlled infusion pump (Orchestra^®^, Fresenius Vial, France). After tracheal intubation, anesthesia was maintained with desflurane (5–6%) in 50% oxygen/air and continuous infusion of remifentanil (2–5 ng/mL of effect-site concentration) to maintain systolic blood pressure within 20% of baseline values. When skin closure was started, the effect-site concentration of remifentanil was reduced and maintained at 1.5 ng/dL until extubation. After emergence from general anesthesia, the patients were transferred to the PACU.

The need for rescue analgesia was evaluated from 0 to 24 h after surgery. In the PACU, intravenous fentanyl (0.4 µg/kg) was administered when NRS was ≥4 or when the patient was in need of pain relief. Administration of fentanyl was repeated until NRS <4 or the patient did not request further pain relief. In the general ward, 30 mg of ketorolac was administered first according to the same rescue analgesic criteria as applied in the PACU. When the effect of ketorolac was not enough, 50 mg of tramadol or 25 mg of meperidine was administered during 24 h after surgery. Total doses of rescue analgesics during 24 h after surgery were recorded and then converted to equianalgesic doses of intravenous fentanyl based on the previously published conversion factors (intravenous fentanyl 100 µg = ketorolac 30 mg = tramadol 100 mg) [22,23]. Total or cumulated rescue analgesic consumption are expressed in intravenous fentanyl equivalents (µg).

### 2.5. Ultrasound-Guided RSB and ICNB

In the pre-RSB group, RSB and ICNB were performed after anesthesia induction and before skin incision. In the post-RSB group, RSB and ICNB were performed after skin closure and before emergence from anesthesia. RSB and ICNB were performed using NextGen LOGIQ e ultrasound console (GE Healthcare, Madison, WI, USA) with a 12 MHz high-frequency linear array transducer.

For the RSB, the ultrasound probe was positioned transversely on the rectus abdominis muscle, below the umbilicus. Guided by real-time ultrasound, a 23-gauge Quincke needle (TaeChang Industrial Co., Gongju, Korea) was inserted in-plane with caution to avoid injury to nearby vessels from medial to lateral direction, until its tip positioned in the plane between the lateral side of rectus abdominis muscle and the posterior rectus sheath. After negative pressure aspiration, 17 mL of 0.25% ropivacaine was administered, and then the block was repeated on the opposite side.

For the ICNB, the ultrasound probe was positioned in the parasagittal axis to get a total view of the pleura and three layers of intercostal muscles (external, internal, and innermost) between two adjacent ribs at a target level. The targets were the right sixth, seventh, and eighth intercostal nerve, determined by the subxiphoid and subcostal port sites. The needle was inserted in-plane from caudal to cephalad direction until its tip positioned between the internal intercostal muscle and the innermost intercostal muscle. After aspiration, 2 mL of 0.25% ropivacaine was injected for each intercostal nerve. The total dose of ropivacaine for RSB and ICNB were the same in all patients of both groups, 40 mL of 0.25% ropivacaine.

### 2.6. Outcome Measures and Data Collection

The primary outcome was total rescue analgesic consumption during 24 h after surgery. Secondary outcomes were cumulated rescue analgesic consumption and postoperative pain scores at 0, 1, 2, 6, 9, 18, and 24 h after surgery. The other outcomes included intraoperative remifentanil consumption, changes in vital signs related to skin incision, the adverse effects of analgesics, and complications associated with RSB and ICNB.

Intraoperative evaluation was performed by the intervention staff. Postoperative evaluation was performed by the second investigator who was blinded to treatment allocation. In the PACU, the first postoperative evaluation of RSB and ICNB was performed. An algometer (Baseline algometer, Baseline^®^, India) was used to induce experimental pressure pain on each of the three trocar sites (below the xiphoid process, right costal arch, and umbilicus). The pressure was applied for 10 s on each trocar site to exclude patients of insufficient RSB or ICNB, which was determined by the threshold of pressure pain lower than 2 kg/cm^2^ pressure [24,25]. Cumulated rescue analgesic consumption and postoperative pain scores were measured at 0, 1, 2, 6, 9, 18, and 24 h after surgery. Postoperative pain scores were assessed using an 11-point numerical rating scale (NRS), in which 0 = no pain and 10 = worst pain imaginable. The side effects of analgesics, such as dizziness, sedation, respiratory depression, nausea, and vomiting were all checked. In addition, the complications associated with RSB and ICNB were evaluated, including pneumothorax and hematoma.

### 2.7. Statistical Analysis

Sample size was calculated based on the results of our previous data, in which the means ± standard deviations of total rescue analgesic consumption during 24 h post-surgery in pre-RSB and post-RSB groups were 170 ± 69 µg and 200 ± 69 µg, respectively. To detect this difference with an alpha of 0.05 (two-sided) and a power of 0.8, 85 patients per group were needed. Allowing for a 15% dropout during the study period, 100 patients were recruited for each group.

Between-group comparisons were evaluated using the Student’s t-test or Mann–Whitney U-test for continuous variables, and the chi-square or Fisher’s exact test for categorical variables, as appropriate. Continuous variables were reported as mean (± standard deviation) or median (interquartile range) and categorical variables as frequency (percentage). A two-tailed *p* < 0.05 was considered significant. Statistical Package for the Social Sciences (SPSS, version 22.0, IBM SPSS Statistics, IBM Corporation, Armonk, NY, USA) was used for data manipulation and statistical analysis.

## 3. Results

**Figure 1:**
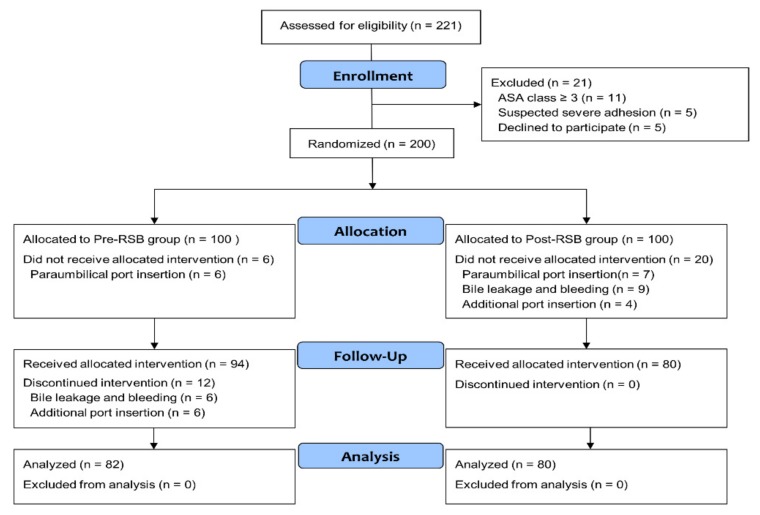
Flowchart of the study population. Abbreviations: ASA, American Society of Anesthesiologists; Pre-RSB, preoperative rectus sheath block; Post-RSB, postoperative rectus sheath block.

Of the 221 patients screened from March 2017 to January 2018, 21 patients were excluded from the study (Figure 1). 200 patients were randomized into two groups (pre-RSB (*n* = 100) and post-RSB (*n* = 100)). 6 patients in the pre-RSB group (paraumbilical port insertion) and 20 in the post-RSB group (paraumbilical port insertion, bile leakage, bleeding, and additional port insertion) did not receive allocated intervention. In addition, 12 patients in pre-RSB group who had bile leakage, bleeding, and additional port insertion during surgery were also excluded. Consequently, 162 patients were included in the final analysis. There were no significant differences in the baseline characteristics between the two groups (Table 1). There was no patient with insufficient RSB or ICNB when assessed by the pressure pain threshold using an algometer in the PACU.

The duration of surgery and anesthesia were not significantly different between the two groups (*p* = 0.275 and *p* = 0.252, respectively), but the pre-RSB group had significantly lower intraoperative remifentanil consumption (µg) than the post-RSB group (451.0 (360.0–540.0) vs. 490.0 (406.5–597.5), *p* = 0.012) (Table 2). Compared with the post-RSB group, the pre-RSB group had no significant changes in the vital signs related to the skin incision.

**Figure 2:**
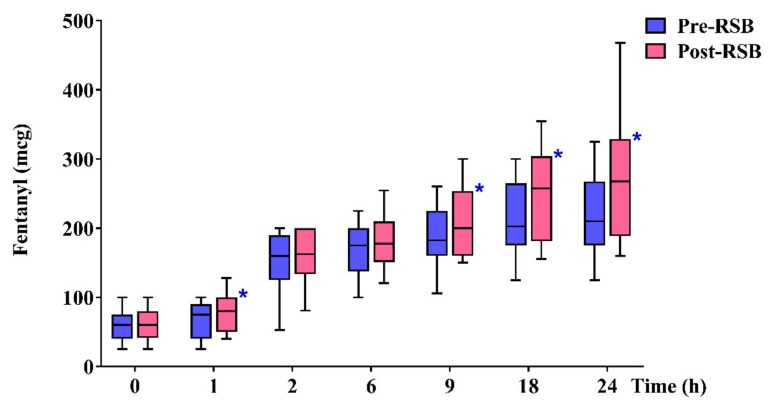
Cumulated rescue analgesic consumption during 24 h after surgery. Data are expressed as median (interquartile range). Horizontal lines, boxes, and error bars represent the median, interquartile range, and 10th and 90th percentile, respectively. * *p* < 0.05. Abbreviations: Pre-RSB, preoperative rectus sheath block; Post-RSB, postoperative rectus sheath block.

Total rescue analgesic consumption during 24 h after surgery (µg) was significantly lower in the pre-RSB group than in the post-RSB group (210.0 (175.0–265.0) vs. 267.5 (192.5–327.5), *p* = 0.020) (Figure 2). Within postoperative 24 h, the cumulated rescue analgesic consumption (µg) was significantly lower in the pre-RSB group than in the post-RSB group at 1, 9, and 18 h after surgery (75.0 (40.0–90.0) vs. 80.0 (50.0–100.0), *p* = 0.023; 182.5 (160.0–225.0) vs. 200.0 (160.0–252.5), *p* = 0.020; and 202.5 (175.0–265.0) vs. 257.5 (182.5–302.5), *p* = 0.002).

**Figure 3:**
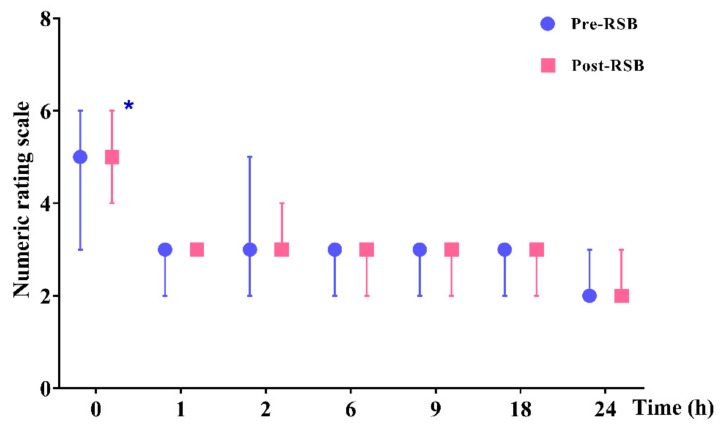
Postoperative pain scores (numerical rating scale 0 to 10) during 24 h after surgery. Data are expressed as median (interquartile range). * *p* < 0.05. Abbreviations: Pre-RSB, preoperative rectus sheath block; Post-RSB, postoperative rectus sheath block.

NRS was significantly lower in the pre-RSB group than in the post-RSB group at 0 h after surgery (5.0 (3.0–6.0) vs. 5.0 (4.0–6.0), *p* = 0.023) (Figure 3). Except at 0 h, the NRS was not significantly different between the two groups throughout the 24 h after surgery. No patient complained of significant side effects related to the analgesics or complications associated with RSB or ICNB.

## 4. Discussion

This study demonstrated that in patients undergoing LC, pre-RSB resulted in significantly lower intraoperative remifentanil consumption and postoperative 24-h analgesic consumption compared with post-RSB. There was no significant difference in the postoperative NRS between the two groups except at 0 h after surgery, when NRS was significantly lower in the pre-RSB group than in the post-RSB group.

Pain relief after LC is an issue of great practical importance. Pain after LC remains the primary reason for delayed hospital stay and prolonged convalesce after surgery [4,26]. In the first 24 h after surgery, classic LC causes moderate to severe postoperative pain [4] with the incisional pain being dominant over the visceral pain, and the port-site wounds remained the most painful region [4,6]. The complexity (incisional, visceral, and referred shoulder pain) and severity of the pain after LC suggest that multimodal analgesic management may be necessary in patients undergoing LC [7,8,9,27].

Multimodal analgesic regimen for patients undergoing LC has been studied. Administration of NSAIDs, COX-2 inhibitors, preoperative single-dose dexamethasone, and port site infiltration of local anesthetics are recommended; the efficacy of gabapentinoids, intraperitoneal instillation of local anesthetics, and TAP block are debatable; and the use of opioids is reserved for rescue analgesia [7,8,9,27]. Notably, infiltration of local anesthetics in all trocar incisions has been recommended as a routine analgesic regimen for LC [27].

Compared with LAI in LC, the analgesic effect of RSB in classic LC is not well proven. However, RSB may provide superior analgesia for the infraumbilical port site which is the main source of pain in the immediate postoperative period, compared with LAI [16,17]. In addition, RSB has been proven to improve the pain after laparoscopic gynecologic surgery and LC, compared with intraincisional or intraperitoneal infiltration of local anesthetic [13,14]. However, there is currently insufficient evidence to support the superiority of RSB over LAI to relieve pain after LC. Further studies are warranted to clarify this issue. Although TAP block has also been evaluated for pain relief after classic LC, its analgesic effect over placebo or LAI has not been clear [12,28,29]. Compared with TAP block, RSB may provide more satisfactory analgesia for incisions in the midline, because the sensory block area of the TAP block is predominantly located lateral to a vertical line through the anterior superior iliac spine [30]. In addition, RSB may provide prolonged blockade of noxious input from the incision site owing to it slower absorption kinetic profile than TAP block [31]. To relieve the somatic pain from the main trocar site (infraumbilical site), we performed bilateral RSB instead of LAI or TAP block.

Surgery-induced central sensitization is triggered by incision, intraoperative trauma, and postoperative inflammatory inputs. Therefore, the ideal preemptive analgesia may be the treatment that prevents the establishment of central sensitization caused by both intraoperative noxious inputs and postoperative inflammatory injuries; the treatment starts before incision and covers both the intraoperative period and the initial postoperative period [18,19].

Several randomized double-blinded studies have presented the preemptive analgesic effect of peripheral nerve blocks [32,33,34]. When the degree of afferent blockade was sufficient and the blockade extended into the initial postoperative period, the analgesic effect of peripheral nerve block lasted beyond the duration of nerve block, resulting in lower pain intensity and/or analgesic consumption for more than 24 h after surgery. To our knowledge, this is the first study aimed to evaluate the preemptive effect of RSB in the patients undergoing classic LC. In this study, pre-RSB resulted in significantly lower analgesic requirements during 24 h post-surgery, compared with post-RSB. Considering that the median duration of LC was 36.0 min in pre-RSB group, the effect of pre-RSB with 0.25% ropivacaine may have well extended into the initial postoperative period. Therefore, both pre-RSB and post-RSB may have reduced pain hypersensitivity owing to postoperative inflammatory input compared with neither pre-RSB nor post-RSB. However, pre-RSB resulted in lower postoperative analgesic requirements than post-RSB because of its additional blockade of the intraoperative noxious input, thus minimizing the development of central sensitization [18,19]. This study suggests the preemptive effect of RSB on acute postoperative pain after LC.

Studies on the preemptive effect of RSB in patients undergoing laparoscopic surgery are limited, and some are contrary to our results. First, Kim et al. reported that bilateral RSB decreased the intensity of superficial pain only during the first hour after robotic cholecystectomy, compared with a placebo group [35]. Second, Jin et al. reported that there was no significant difference in the pain, analgesic requirements, or time to first rescue analgesic after transabdominal gynecological surgery between the pre-RSB and post-RSB groups [36]. However, in both studies, only female patients were included, and the ports or incisional sites were limited in the lower abdomen which usually involves less postoperative pain than the upper abdomen. Opposite to our study, RSB was performed in the lower abdomen below the arcuate line, and the sufficiency of blockade was not confirmed in both studies. In the first study of robotic cholecystectomy, only the main port site (one 12 mm) not smaller port sites (two 7 mm) was blocked. In the second study of transabdominal gynecological surgery, bilateral single-shot RSB may have been insufficient to cover intraoperative and initial postoperative afferent input from the median 9 cm incision site. Furthermore, drug leakage, which was always detected in the pre-RSB group by a surgeon’s incision, may have attenuated the effect of preoperative block. Therefore, our results may not be directly compared with the above studies.

This study has a limitation. Including a placebo group (neither pre-RSB nor post-RSB) may have been a more reasonable approach to demonstrate the preemptive effect of RSB in the patients undergoing LC [19]. In that way, the efficacy of pre-RSB or post-RSB on postoperative pain scores over the placebo group may have been detected. The relative efficacy of post-RSB on postoperative analgesic requirements over the placebo group may have also been detected.

## 5. Conclusions

In conclusion, preoperative RSB lowered the analgesic requirements during the intraoperative and postoperative 24-h period in patients undergoing LC, compared with postoperative RSB. The timing of RSB may be of clinical significance to preempt postoperative pain following LC.

## Figures and Tables

**Table 1:** Baseline characteristics of the study participants.

	Pre-RSB Group (*n* = 82)	Post-RSB Group (*n* = 80)	*p*-Value
Age	57.0 (47.0–63.0)	55.0 (42.0–64.0)	0.327
Sex (female)	43 (52.4%)	45 (56.2%)	0.742
Body mass index (kg/m^2^)	24.6 (22.9–26.2)	24.2 (21.9–26.3)	0.469
ASA PS			0.593
I	32 (39.0%)	27 (33.8%)	
II	50 (61%)	53 (66.2%)	
Hypertension	7 (8.5%)	11 (13.8%)	0.420
Diabetes mellitus	20 (24.4%)	22 (27.5%)	0.785
Pathologic diagnosis			0.955
Acute cholecystitis	63 (76.8%)	60 (75.0%)	
Chronic cholecystitis	11 (13.4%)	12 (15.0%)	
Gallbladder polyp	8 (9.8%)	8 (10.0%)	
Biliary drainage			0.702
None	69 (84.1%)	70 (87.5%)	
ENBD	9 (11.0%)	8 (10.0%)	
PTBD	4 (4.9%)	2 (2.5%)	

Data are expressed as median (interquartile range) or numbers (%), as appropriate. Abbreviations: pre-RSB, preoperative rectus sheath block; post-RSB, postoperative rectus sheath block; ASA PS, American Society of Anesthesiologists Physical Status; ENBD, endoscopic nasobiliary drainage; PTBD, percutaneous transhepatic biliary drainage.

**Table 2:** Intraoperative data.

	Pre-RSB Group (*n* = 82)	Post-RSB Group (*n* = 80)	*p*-Value
MBP change after incision (%)	0.0 (−2.9–2.9)	10.6 (5.4–18.9)	<0.001
HR change after incision (%)	0.0 (−2.1–1.7)	5.6 (1.4–12.8)	<0.001
Duration of surgery (min)	36.0 (30.0–45.0)	40.0 (30.0–47.5)	0.275
Duration of anesthesia (min)	70.0 (60.0–80.0)	70.0 (65.0–85.0)	0.252
Intraoperative remifentanil requirement (µg)	451.0 (360.0–540.0)	490.0 (406.5–597.5)	0.012

Values are expressed as median (interquartile range). Abbreviations: pre-RSB, preoperative rectus sheath block; post-RSB, postoperative rectus sheath block; MBP, mean blood pressure; HR, heart rate.
